# OsNAC2 Is Involved in Multiple Hormonal Pathways to Mediate Germination of Rice Seeds and Establishment of Seedling

**DOI:** 10.3389/fpls.2021.699303

**Published:** 2021-07-23

**Authors:** Jiangtao Yu, Chanjuan Mao, Qun Zhong, Xuefeng Yao, Peng Li, Chunming Liu, Feng Ming

**Affiliations:** ^1^Shanghai Key Laboratory of Plant Molecular Sciences, College of Life Sciences, Shanghai Normal University, Shanghai, China; ^2^The Biotechnology Research Institute, Shanghai Academy of Agricultural Sciences, Shanghai, China; ^3^Key Laboratory of Plant Molecular Physiology, Institute of Botany, Chinese Academy of Sciences, Beijing, China; ^4^The Biotechnology Research Institute, Shanghai Academy of Agricultural Sciences, Shanghai, China; ^5^Institute of Crop Sciences, Chinese Academy of Agricultural Sciences, Beijing, China

**Keywords:** rice, OsNAC2, ethylene, abscisic acid, seed germination, seedling growth

## Abstract

The germination of seeds and establishment of seedling are the preconditions of plant growth and are antagonistically regulated by multiple phytohormones, e.g., ethylene, abscisic acid (ABA), and gibberellic acid (GA). However, the interactions between these phytohormones and their upstream transcriptional regulation during the seed and seedling growth in rice remain poorly understood. Here, we demonstrated a rice NAC (NAM-ATAF-CUC) transcription factor, OsNAC2, the overexpression of which increases the ethylene sensitivity in rice roots during the seedling period. Further study proved that OsNAC2 directly activates the expressions of *OsACO* and *OsACO3*, enhancing ethylene synthesis, and then retards seedling establishment. Moreover, OsNAC2 delays the germination of seeds and coleoptile growth through the ABA pathway instead of the ethylene and GA pathway, by targeting the promoters of *OsNCED3*, *OsZEP1*, and *OsABA8ox1*. We also found that OsNAC2 regulates downstream targets in a time-dependent manner by binding to the promoter of *OsKO2* in the seedling period but not in the germination stage. Our finding enriched the regulatory network of ethylene, ABA, and GA in the germination of rice seeds and seedling growth, and uncovered new insights into the difference of transcription factors in targeting their downstream components.

## Introduction

Rice (*Oryza sativa* L.) is one of the most important cereal foods worldwide. During its life cycle, the germination of seeds and seedling formation are always the preconditions of plant growth and development. The germination of seeds, starting from water absorption till radicle protrusion, is a critical phase that provides rice plants an essential opportunity to survive in a hostile environment and to establish new seedlings (Bewley, 1997; Rajjou et al., 2012). Seedling establishment is a close and continuous biological process occurring post germination, which is also one of the important factors affecting the rice yield (Pyo et al., 2010).

The germination of seeds is a complex physiological process that can be controlled by various phytohormones, e.g., ethylene, abscisic acid (ABA), gibberellic acid (GA), brassinosteroids, auxin, and cytokinin. Among them, ABA and GA are reported to play the major roles. A major conclusion, since 1980s, is that ABA negatively regulates the germination of seeds, while GA promotes this event (Bewley, 1997; Nambara and Marion-Poll, 2005; Tognacca and Botto, 2021). Several components modulating the ABA and GA pathways in seeds have been identified in *Arabidopsis* and rice. The two type-2C protein phosphatases, namely, Abelson interactor 1 (ABI1) and Abelson interactor 2 (ABI2), negatively regulate ABA signaling, and their mutants confer increased germination rate (Rodriguez et al., 1998; Assmann et al., 2000). Rice *Delayed Seed Germination 1* (*OsDSG1*) gene, an ortholog of *AtAIP2*, directly binds to *OsABI3*, inhibits expressions of ABA-signaling genes, and finally promotes the germination of seeds (Park et al., 2010). Antagonistic to ABA, GA has been reported to function in boosting germination and radical protrusion. Rice *GERMINATION DEFECTIVE 1* (*OsGD1*) acts as a transcriptional repressor in GA metabolism and, further, regulates the germination of seeds (Guo et al., 2013). *ent-kaurene oxidase 1* (*OsKO1*) functions in the biosynthesis of GA, mutations of which inhibited germination in rice (Zhang et al., 2020). Besides ABA and GA, ethylene is generally recognized to promote the germination of seeds and acts as an antagonist to ABA in seeds of *Arabidopsis* and other species (Ghassemian et al., 2000; Kucera et al., 2005; Linkies et al., 2009; Matilla and Matilla-Vazquez, 2008). The *ethylene-insensitive ethylene response 1* (*etr1*) mutant exhibits a poor germination rate probably due to the accumulation of ABA (Chiwocha et al., 2005). The constitutive ethylene-response mutant *ctr1* showed a weakened ABA response in the germination of seeds (Beaudoin et al., 2000). However, it remains largely unknown how cross talk among ABA, GA, and ethylene modulates the germination of seeds, and their upstream components still need to be investigated.

In contrast with the situation in the germination of seeds, the seedling growth is inhibited by ethylene (Etheridge et al., 2006). It has been reported that ethylene plays essential roles as in etiolated *Arabidopsis* seedlings called the triple response, while in dark-grown rice seedlings called the double response, including coleoptile promotion and root inhibition (Yang et al., 2015). In rice, several *Arabidopsis* homologous genes have also been identified in ethylene signaling. Mutation of ethylene receptor gene *ETHYLENE RESPONSE SENSOR1* (*OsERS1*) leads to enhanced ethylene sensitivity in dark-grown seedlings and delayed root growth (Ma et al., 2014). The overexpression of *OsRTH* (*RICE RTE1 HOMOLOG*) reduces the ethylene sensitivity and prevents the root development and the coleoptile elongation (Zhang et al., 2012). *OsEIN2* antisense plants were dysgonic and their shoot growth was restrained, with reduced expressions of ethylene-responsive genes (Jun et al., 2004). The overexpression of *OsEIL1* in plants showed enhanced ethylene response, with short roots and shoots (Mao et al., 2006). Some rice *MHZ* genes were reported to take part in ethylene response and seedling growth (Yang et al., 2015). MHZ4, homologous of *Arabidopsis* ABA4, inhibits rice root growth due to the accumulation of ABA (Ma et al., 2014). *MHZ1*, encoding a kinase protein, positively regulates ethylene responses in root by interacting with ethylene receptor OsERS2 (Zhao et al., 2020). Besides the signaling pathway, the function of genes in ethylene metabolism also affects seedling growth. Mutants of *ETHYLENE OVERPRODUCER* [*eto*(s)] developed short roots due to an enhanced biosynthesis of ethylene (Wang et al., 2012). Both 1-aminocyclopropane-1-carboxylic acid (ACC) synthase 5 (ACS5/ETO2) and ACS8 function in ethylene synthesis and mediate hook development (Gallego-Bartolomé et al., 2011). However, the upstream network of ethylene signaling and metabolism remains to be investigated further.

Transcriptional regulation appears central in plant growth, and transcription factors function in the modulation of the whole plant life cycle. The involvement of NAC (NAM-ATAF-CUC) transcription factors in plant developmental programs, including embryonic, floral, and vegetative development, is well documented (Aida et al., 1997; Olsen et al., 2005; Xie et al., 2000). During seed and seedling growth, auxin is associated with NAC transcription factor *NTM2* through *IAA30* gene, and it regulates the *NTM2*-mediated salt signaling pathway and delays the germination of seeds of *Arabidopsis thaliana* (Park et al., 2011). *NTL8* delayed the germination of seeds under high-salt conditions by GA-regulating salt signals in *A. thaliana* (Kim et al., 2008). Additionally, the DELLA protein RGL2 repressed the activation of the *EXPA2* promoter by NAC25/NAC1L, and it regulates endosperm cell expansion and plays a vital role in *A. thaliana* from seed to seedling (Sánchez-Montesino et al., 2019). However, the molecular characterization of NAC family regarding their effects on the germination of seeds and seedling growth in rice remains largely unknown.

Earlier, we reported that a rice NAC family transcription factor, OsNAC2, is involved in the development of rice and abiotic response by binding to different downstream targets (Chen et al., 2015; Mao et al., 2017, 2018, 2020; Shen et al., 2017). In this study, we revealed that OsNAC2 negatively regulates the germination of rice seeds and seedling growth. Further study proved that OsNAC2 increases the ethylene sensitivity and promotes the biosynthesis of ethylene by directly binding to *OsACO* and *OsACO3* promoters. In addition, OsNAC2 inhibits the germination of rice seeds probably through the ABA pathway instead of the ethylene and the GA pathways. We also evidenced that OsNAC2 regulates its downstream target *OsKO2* in a time-dependent manner. Our findings enrich the regulatory network of ethylene, ABA, and GA in the germination of rice seeds and seedling growth and uncover new insights into the difference of transcription factors in targeting their downstream components.

## Results

### Overexpression of OsNAC2 Leads to Enhanced Ethylene Response and Synthesis

**FIGURE 1:**
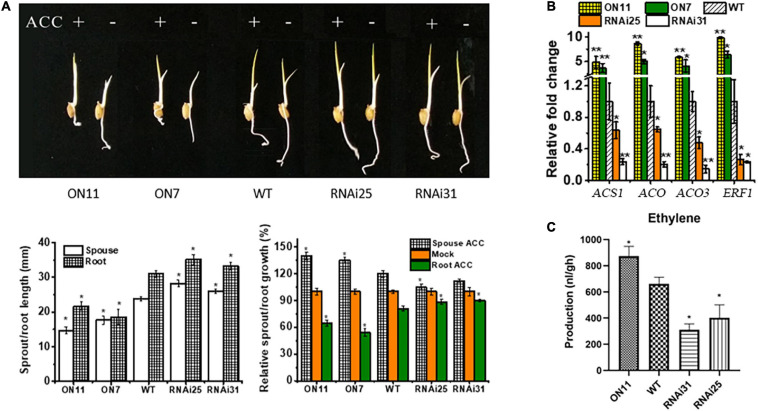
Effects of *OsNAC2* on the synthesis and response of ethylene in the *OsNAC2*-transgenic lines at the seedling formation stage. **(A)** Phenotype of *OsNAC2*-transgenic seedlings treated with 30 μm 1-aminocyclopropane-1-carboxylic acid (ACC) for 3 days under dark. **(B)** Expressions of ethylene synthesis and response genes in the wild-type (WT) and *OsNAC2-*transgenic seedlings. The relative mRNA level was calculated using the ΔΔC_T_ method from triplicate data. *OsActin* was used as internal control to normalize the various samples with the same amount of plant RNA. **(C)** Ethylene production of WT and transgenic seedlings. Data are presented as the mean ± standard error of at least three biological replicates. Asterisks indicate significant differences between treatment and control by *t*-test. *p < 0.05, **p < 0.01, and **p < 0.001.

In previous and current studies, we demonstrated that OsNAC2 plays significant roles in multiple biological functions, e.g., plant height (Chen et al., 2015), senescence (Mao et al., 2017), abiotic stress tolerance (Shen et al., 2017), and programmed cell death (Mao et al., 2018); most of the physiological processes occurred in rice seedling stage. Compared with all the phenotypes, we concluded that OsNAC2 inhibited rice seedling growth, with weakened shoot and root lengths. It has been generally reported that the seedling growth is inhibited by ethylene (Nemhauser et al., 2006). Thus, we wondered whether OsNAC2 is involved in the ethylene pathway to regulate seedling growth. To validate this hypothesis, we first assessed the phenotype of different *OsNAC2* transgenic lines and the wild type (WT) with or without treatment with ethylene. Compared with the WT, the seedlings of the *OsNAC2*-overexpressing lines, namely, ON7 and ON11, showed shorter sprout and root, while the *OsNAC2*-RNAi lines (i.e., RNAi25 and RNAi31) showed the opposite phenotype (Figure 1A). Under the treatment with ACC, the inhibition rate of root and the promotion rate of sprout were significantly higher in the overexpression lines, which strongly suggested that OsNAC2 enhanced the ethylene sensitivity in both rice root and sprout (Figure 1A). When treated with aminoethoxyvinylglycine (an inhibitor of the biosynthesis of ethylene), the suppression of root and sprout growth was obviously compromised in the *OsNAC2*-overexpressing lines (Supplementary Figure 1). In addition, the expressions of ethylene synthesis and response genes, namely, *OsACS1, OsACO, OsACO3*, and *OsERF1*, were upgraded to approximately 3–10-folds in the WT in the *OsNAC2*-overexpressing lines (Figure 1B). Moreover, the ethylene contents of the *OsNAC2*-RNAi lines were 401.7 and 311.0 nl/g.h, respectively, which were significantly lower than that of WT (695.94 nl/g.h), while the *OsNAC2*-overexpressing line ON11 showed the highest ethylene emission (873.22 nl/g.h) (Figure 1C). These results indicated that *OsNAC2* may affect the formation of rice seedlings by strengthening the ethylene response and synthesis.

### OsNAC2 Binds Directly to the OsACO and OsACO3 Promoters

**FIGURE 2:**
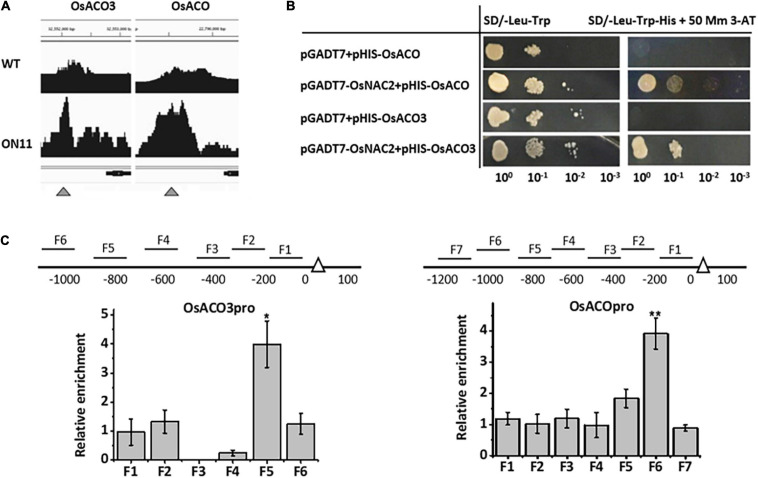
OsNAC2 directly regulates ethylene synthesis–related genes. **(A)** Binding peaks of *OsACO* and *OsACO3* promoters in the chromatin immunoprecipitation (ChIP)-sequencing assay. Black peaks represent the sequence hits in each gene region. The peak height corresponds to the extent of the binding in a particular region. Bars above the peaks indicate the distance from the ATG start codon of each gene. The scale was 500 bp. The black bar under the peaks represents the coding sequence of each gene, with arrows indicating the direction. **(B)** Interaction between OsNAC2 and *OsACO* and *OsACO3* promoters by using yeast one-hybrid assay. The yeast cells were grown on SD/-Leu/-Trp/-His/ + 30 mM 3-amino-1,2,4-triazole medium. **(C)** ChIP–quantitative polymerase chain reaction (qPCR) assay to confirm the binding of OsNAC2 to *OsACO* and *OsACO3* promoters. Total protein extracted from 35S:OsNAC2-mGFP transgenic seedlings were immunoprecipitated with an anti–green fluorescent protein (GFP) antibody. Fragmented genomic DNA was eluted from the protein–DNA complexes and subjected to the quantitative polymerase chain reaction (qPCR) analysis. The long black bars represent promoter regions for which we designed primers. The numbers under the bar show the distance from the ATG start codon. Short bars represent the corresponding region of each pair of primers on the promoter. Error bars represent the standard error of three biological replicates. *p < 0.05 and **p < 0.01.

To further identify whether OsNAC2 directly regulated genes involved in ethylene metabolism or signaling, we performed a chromatin immunoprecipitation (ChIP)-sequence to scan the promoter area in genes of interest. The enrichment peaks were detected in two candidate genes, namely, *OsACO* and *OsACO3*, which function in ethylene production (Figure 2A). Furthermore, the yeast one-hybrid assay showed that OsNAC2 could activate the HIS3 reporter gene, driven by the promoters of *OsACO* and *OsACO3* (Figure 2B). In addition, the ChIP–quantitative polymerase chain reaction (qPCR) assay was conducted to test the interaction between OsNAC2 and its candidate targets. The result showed that F5 and F6 fragments of the promoters of *OsACO* and *OsACO3* were obviously enriched and immunoprecipitated by anti-green fluorescent protein (GFP) antibody, respectively, compared with the negative control (Figure 2C). Taken together, our data indicated that OsNAC2 is involved in the ethylene synthesis pathway through targeting the promoters of *OsACO* and *OsACO3*.

### OsNAC2 Delays Germination of Seeds and Coleoptile Growth

**FIGURE 3:**
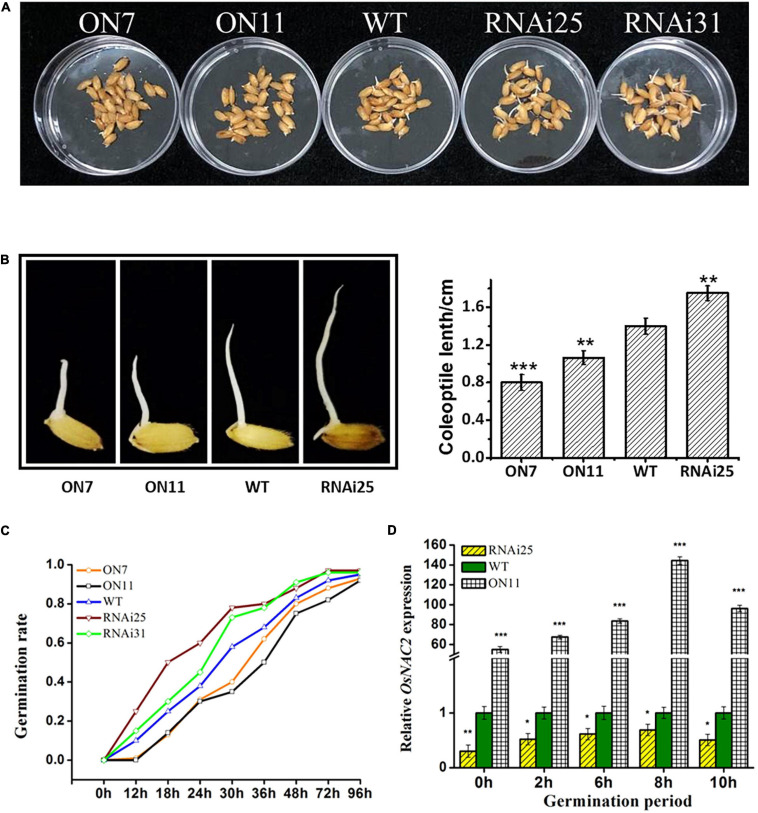
Effects of OsNAC2 on the germination of seeds and coleoptile of *OsNAC2*-transgenic plants. **(A)** Germination phenotype of wild-type (WT) and *OsNAC2*-transgenic seeds under normal conditions of germination. **(B)** Coleoptile length of WT and *OsNAC2-*transgenic seeds. The experiment was conducted at least three times, with 50 seeds in each Petri dish. **(C)** The germination rate of WT and *OsNAC2-*transgenic seeds. The experiment was conducted at least three times, with 50 seeds in each Petri dish. **(D)** Expression pattern of *OsNAC2* during the germination period in the WT, *OsNAC2-overexpressing* (ON11), and *OsNAC2-knockdown* (RNAi25) transgenic seeds. Data are presented as the mean ± standard error of at least three biological replicates. Asterisks indicate significant differences between treatment and control by *t*-test. *p < 0.05, **p < 0.01, and ***p < 0.001.

Besides its function in seedling growth, ethylene has been reported to promote the germination of seeds (Wilson et al., 2014). Since OsNAC2 increased the ethylene synthesis and response, we speculated that whether OsNAC2 affects the germination of rice seeds. To our surprise, the germination rate until 72 h was significantly lower in the *OsNAC2*-overexpressing lines, while higher in RNAi plants, compared with the WT (Figures 3A,C). The length of coleoptile is also an indicator that reflects the germination condition. Thus, we further measured the coleoptile length of the five different *OsNAC2* transgenic lines. The result showed that the coleoptile of the *OsNAC2-overexpressing* lines was significantly suppressed, with the lengths of 0.79 and 1.05 cm, compared with the WT, while it was obviously promoted in the *OsNAC2-knockdown* lines (Figure 3B). To further identify the biological function of OsNAC2 in the germination of seeds, its mRNA level was detected by using qPCR within 16 h germination. We found that *OsNAC2* expression was obviously induced 2 h after germination, and it reached the peak at 8 h and then gradually decreased (Figure 3D). Additionally, the OsNAC2 transcript was significantly higher in the OsNAC2-ovexpressing line ON11, while it markedly decreased in the OsNAC2-RNAi line RNAi25 (Figure 3D). Taken together, these results suggested that OsNAC2 inhibits the germination of seeds and coleoptile growth.

### OsNAC2 Inhibits Germination of Rice Seeds Mainly Through the ABA Pathway

**FIGURE 4:**
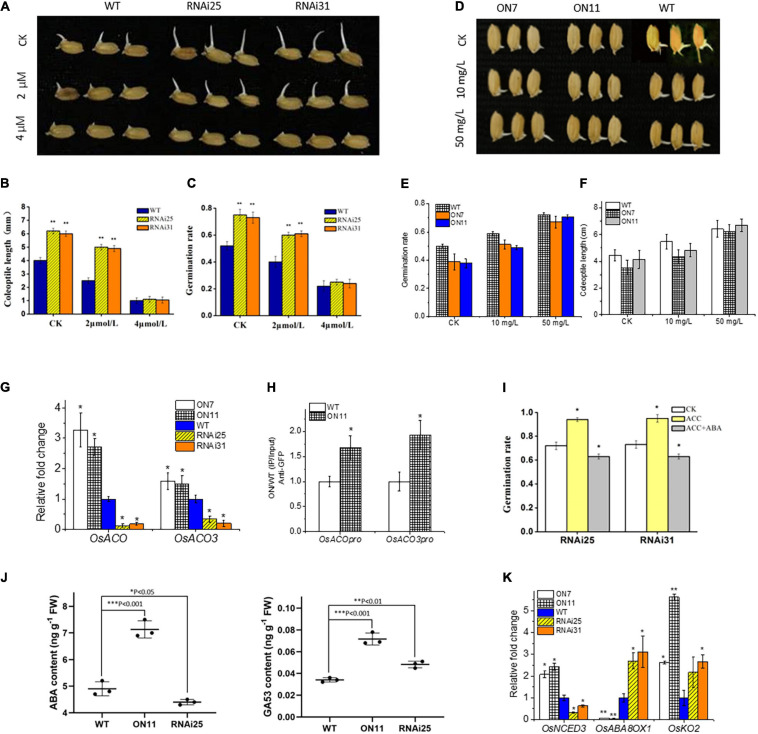
Effects of OsNAC2 on the ethylene and the abscisic acid (ABA) pathways at the stage of the germination of seeds. **(A)** Germination phenotype of the wild-type (WT) and *OsNAC2-knockdown* transgenic seeds treated with 2 or 4 μmol/L of ABA for 24 h. **(B)** Coleoptile length of the WT and *OsNAC2-knockdown* transgenic seeds treated with 2 or 4 μmol/L of ABA for 24 h. **(C)** The germination rate of the WT and *OsNAC2-knockdown* transgenic seeds treated with 2 or 4 μmol/L of ABA for 24 h. **(D)** Germination phenotype of the WT and *OsNAC2*-overexpressing seeds treated with 10 or 50 mg/L of ABA biosynthesis inhibitor fluridone for 24 h. **(E)** The germination rate of the WT and *OsNAC2*-overexpressing seeds treated with 10 or 50 mg/L of ABA biosynthesis inhibitor fluridone for 24 h. **(F)** Coleoptile length of WT and *OsNAC2*-overexpressing seeds treated with 10 or 50 mg/L of ABA biosynthesis inhibitor fluridone for 24 h. **(G)** Expressions of *OsACO* and *OsACO3* in the WT and *OsNAC2*-transgenic seeds during germination. **(H)** Chromatin immunoprecipitation–quantitative polymerase chain reaction (ChIP–qPCR) assay of OsNAC2 on the promoters of *OsACO* and *OsACO3*. **(I)** The germination rate of the WT and *OsNAC2-knockdown* transgenic seeds treated with 30 μmol/L of 1-aminocyclopropane-1-carboxylic acid (ACC) or 2 μmol/L of ABA and 30 μmol/L of ACC for 24 h. **(J)** ABA and GA production in OsNAC2-transgenic seeds. The rice seeds (RNAi25, ON11, and WT) were immersed in pure water at 30 and 37°C, respectively, for 24 h. **(K)** Expressions of ABA and GA metabolism–related genes in the WT and *OsNAC2*-transgenic seeds. Error bars represent the standard error of three biological replicates. *p < 0.05, **p < 0.01, and *p < 0.001.

From the above data, we recognized that OsNAC2 enhanced ethylene synthesis and response, and repressed the germination of seeds and coleoptile growth (Figure 3), which were promoted by ethylene in most studies that involved rice (Yang et al., 2015). This contradiction provided a possibility that OsNAC2 might modulate ethylene synthesis in the seedling period but not in the germination stage. Thus, we tested the relationship between OsNAC2 and *OsACO*s by using qPCR and ChIP–qPCR in the germination of seeds. However, the results showed that expressions of *OsACO* and *OsACO3* were also upregulated and the specific fragments in the promoters of *OsACO* and *OsACO3* were enriched in the *OsNAC2*-overexpressing lines (Figures 4F,G), which suggested that OsNAC2 still functions in the germination of seeds through the ethylene pathway. Taken together, we supposed that some other phytohormones may be involved in this process, of which ABA and GA remain as two major germination regulators. According to our expectation, the germination rate and coleoptile growth were obviously restrained in the *OsNAC2*-RNAi lines when treated with ABA and even similar to that of the WT at 4 μM ABA treatment (Figures 4A–C). In addition, the inhibition of germination and coleoptile growth was greatly compromised in the OsNAC2-overexpressing lines under the treatment with 50 mg/L of ABA inhibitor fluridone (Figures 4D–F and Supplementary Figure 2). However, the inhibition of germination and coleoptile growth could not be compromised in the OsNAC2-overexpressing lines after the treatment with GA (Supplementary Figure 3). Moreover, when treated with GA inhibitor paclobutrazol, the suppression rate of coleoptile growth was not higher in the OsNAC2-RNAi lines, compared with WT (Supplementary Figure 2). To further investigate the interplay between ethylene and ABA in the control of rice germination, 2 μM ABA was used in the complementation assay. In the presence of 30 μM ACC (precursor of ethylene), the application of 2 μM of ABA largely reverses the effect of ethylene on germination of OsNAC2-RNAi plants (Figure 4I), indicating that ABA may play a more important role in the regulation of OsNAC2 in the germination of rice seeds.

Additionally, the transcription level of the key ABA biosynthetic gene *OsNCED3* was significantly upregulated in the *OsNAC2*-overexpressing lines, whereas *OsABA8ox1*, an ABA catabolism gene, was downregulated (Figure 4K). Moreover, the ABA content was dramatically higher in the *OsNAC2*-overexpressing lines, while lower in the *OsNAC2*-knockdown lines (Figure 4J). However, the expression of GA synthetic gene *OsKO2* and GA content was upregulated in both the *OsNAC2*-overexpressing lines and the *OsNAC2*-knockdown lines. We speculated that *OsKO2* may not be affected by *OsNAC2* (Figures 4J,K). Thus, all these results confirmed that OsNAC2 delays the germination of rice seeds mainly through strengthening the synthesis of ABA, instead of ethylene and GA.

### OsNAC2 Regulates Downstream Targets in a Time-Dependent Manner

**FIGURE 5:**
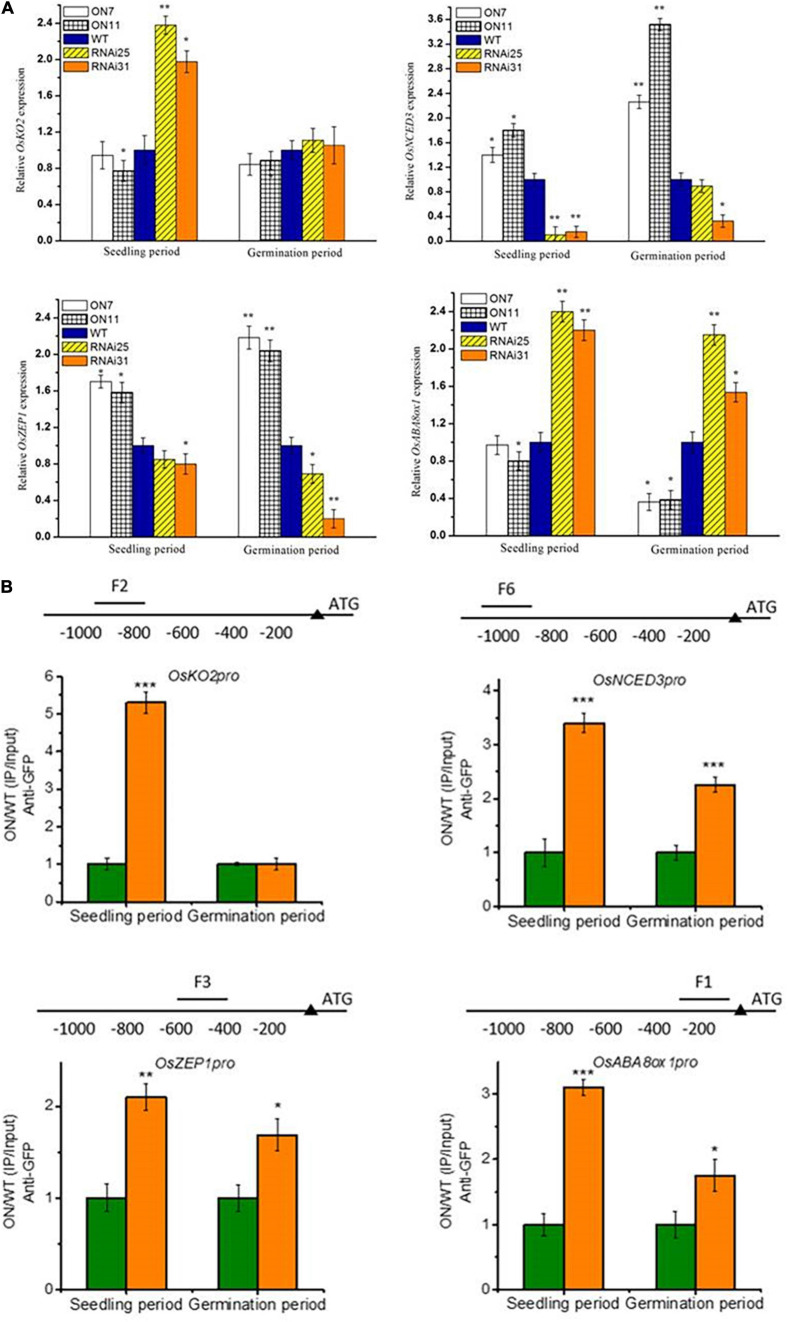
The otherness of OsNAC2 in regulating multiple downstream targets. **(A)** Expressions of abscisic acid (ABA) biosynthetic (*OsNCED3* and *OsZEP1*) and catabolic genes (*OsABA8ox1*) and gibberellic acid (GA) biosynthetic genes (*OsKO2*) in *OsNAC2*-transgenic seeds and seedlings. **(B)** Chromatin immunoprecipitation–quantitative polymerase chain reaction (ChIP–qPCR) assays of OsNAC2 on the promoters of *OsABA8ox1*, *OsNCED3*, *OsZEP1*, and *OsKO2*. Total protein extracted from 35S:OsNAC2-mGFP transgenic seeds were immunoprecipitated with an anti–green fluorescent protein (GFP) antibody. Fragmented genomic DNA was eluted from the protein–DNA complexes and subjected to the qPCR analysis. The long black bars represent promoter regions for which we designed primers. The numbers under the bar show the distance from the ATG start codon. Short bars represent the corresponding region of each pair of primers on the promoter. Error bars represent the standard error of three biological replicates. *p < 0.05, **p < 0.01, and *p < 0.001.

In our earlier study, OsNAC2 binds to the promoter of *OsKO2* and ABA metabolic–related genes (i.e., *OsABA8ox1, OsZEP1*, and *OsNCED3*) to restrain plant height and accelerate leaf senescence of rice seedling, respectively (Chen et al., 2015; Mao et al., 2017). The above result shows that OsNAC2 affected the expressions of ABA-related genes but not GA-related genes during germination, which was consistent with the content of ABA and GA (Figures 4J,K). Thus, it is proposed that OsNAC2, acting as a transcription factor, might target different downstream genes in different physiological processes. Through the qPCR preliminary verification, we found that OsNAC2 can upregulate the expressions of *OsNCED3* and *OsZEP1* and downregulate the expression of *OsABA8ox1* in the germination stage of seeds and in the establishment stage of seedling. However, the expression of *OsKO2* is not regulated by OsNAC2 during the germination period (Figure 5A). Moreover, ChIP is an ideal approach to confirm the interaction between DNA and protein *in vivo*. Further demonstrated by ChIP–qPCR, we found that *OsNCED3*, *OsZEP1*, and *OsABA8ox1* promoter regions were enriched in the germination period and seedling period in the *OsNAC2*-overexpressing lines, immunoprecipitated by the anti-GFP antibody. However, OsNAC2 did not bind to the promoter of *OsKO2* in the germination stage, which is consistent with the fact that the gene expression of *OsKO2* is not regulated by OsNAC2 during the germination period (Figure 5B). These results implicated that OsNAC2 can differently regulate downstream targets in different rice growth and development periods.

### OsACO and OsABA8ox1 Function Downstream of OsNAC2

**FIGURE 6:**
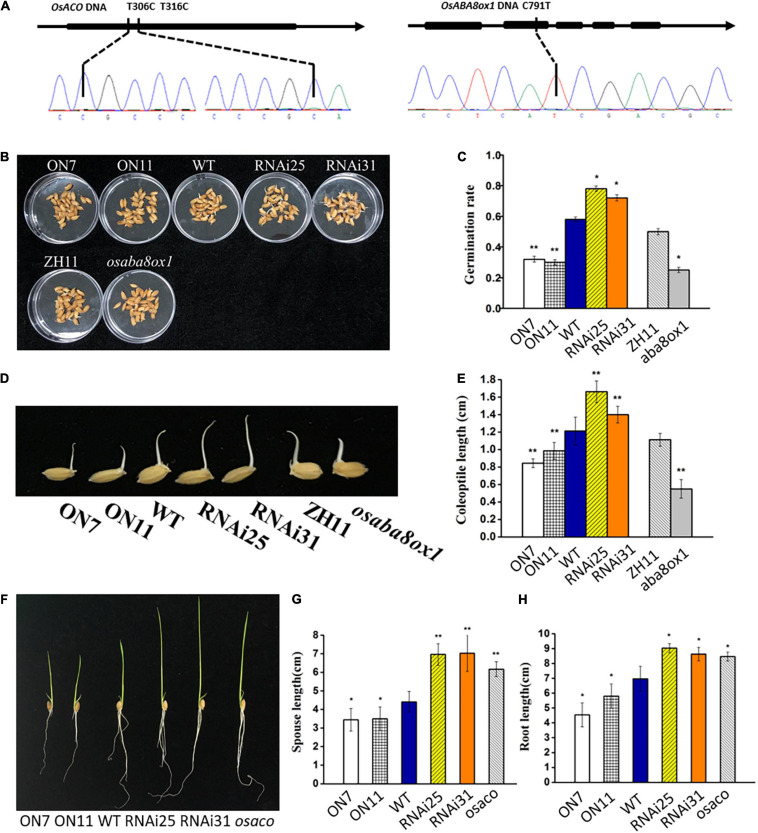
Verification and phenotype and of the *osaco* and *osaba8ox1* lines obtained by using tilling technology. **(A)** Verification of the *osaco* and *osaba8ox* lines by polymerase chain reaction (PCR)-based sequencing. **(B)** Germination phenotype of the wild-type (WT) and *OsNAC2*-transgenic seeds and *osaba8ox1* mutant under normal conditions germination. **(C)** The germination rate of the WT and *OsNAC2*-transgenic seeds and *osaba8ox1* mutant under normal conditions germination. The experiment was conduct at least three times, with 50 seeds in each Petri dish. **(D)** Coleoptile phenotype of the WT and *OsNAC2*-transgenic seeds and *osaba8ox1* mutant under normal conditions germination. **(E)** Coleoptile length of the WT and *OsNAC2*-transgenic seeds and the *osaba8ox1* mutant under normal conditions germination. **(F)** Seedling phenotype of the WT and *OsNAC2*-transgenic seedlings and the *osaco* mutant. **(G)** Sprout length of the WT and *OsNAC2*-transgenic seedlings and the *osaco* mutant. **(H)** Root length of the WT and *OsNAC2*-transgenic seedlings and the *osaco* mutant. Error bars represent the standard error of three biological replicates. *p < 0.05, **p < 0.01, and *p < 0.001.

To further test whether OsNAC2 regulates the germination of seeds and seedling growth *via* its effects on the ABA and ethylene pathways, tilling technology was applied to knock out the endogenous *OsACO* or *OsABA8ox1* in a WT line (ZH11). *osaco* and *osaba8ox1*, with T306C, T316C, and C791T mutations in the target site, respectively, were chosen for further analysis (Figure 6A). This result showed that the germination rate and coleoptile growth of the *osaba8ox1* mutant were obviously inhibited than those of the WT (Figures 6B–E), while the sprout and root length in *osaco* mutant increased about 2 cm than those of the WT (Figures 6F–H). Overall, these data strongly suggested that *OsACO* or *OsABA8ox1* functions downstream of OsNAC2 in the germination of rice seeds and seedling growth.

## Discussion

Ethylene, a simple gaseous phytohormone, regulates distinct physiological processes in plant growth and stress response (Morgan and Drew, 1997; Van et al., 2006). The molecular pathway of ethylene signaling has been well characterized based on triple-response mutants in *A. thaliana* (Huang et al., 2003; Wen et al., 2012). Consistently, ethylene inhibits the growth of both roots and coleoptiles in some monocotyledonous plants, e.g., wheat, maize, and *Brachypodium distachyon* (Yang et al., 2015). In rice, etiolated seedlings showed the double response when treated with ethylene, promoting the coleoptile growth, while delaying the root elongation (Ma et al., 2013). Consistently, we also confirmed the double response of ethylene in rice (Figure 1A). The promotional effect of ethylene on rice coleoptiles is probably due to its water-saturated soil life (Magneschi and Perata, 2009). The molecular components involved in the ethylene-response pathway have also been reported in rice. *MHZ7* positively regulates ethylene response, and its mutant shows ethylene-insensitive phenotype in both root and coleoptile (Ma et al., 2013). The *mhz1* mutant is insensitive to ethylene only in the root (Zhang et al., 2020). In our study, under the treatment with ACC, the inhibition rate of root growth and the promotion rate of coleoptile were significantly higher in the overexpression lines, which strongly suggested that OsNAC2 enhanced ethylene sensitivity in both rice root and coleoptile (Figures 1, 3). It should be noted that OsNAC2 did not lead to longer coleoptile of the enhanced ethylene response phenotype without ethylene treatment, which is inconsistent with MHZ7 or MHZ1. In addition, OsNAC2 accelerates the ethylene production by directly binding to the promoters of *OsACO* and *OsACO3* (Figure 2). These results indicate that OsNAC2 was a novel regulatory element of ethylene signaling and a metabolism component to modulate rice seedling growth.

It became evident that interactions among different phytohormones, including GA, ABA, and ethylene, play vital roles in the germination of seeds. Molecular mechanisms of the GA–ABA interactions in seed growth have been well investigated (Bethke et al., 2007; Piskurewicz et al., 2008; Zhang et al., 2020). Unlike the GA–ABA interaction, interactions between ethylene and ABA in regulating the germination of seeds remain unclear. The seeds of *etr1* mutant showed a poor germination rate partly due to the accumulation of ABA (Chiwocha et al., 2005). Another ethylene-response mutant *ctr1* acts as an enhancer, while *ein2* acts as a suppressor of ABA-insensitive mutant *abi1-1* (Beaudoin et al., 2000). Beaudoin et al. (2000) also revealed the promoted effect of endogenous ethylene on the germination of seeds probably due to reduced ABA sensitivity. In our study, although OsNAC2 still directly increased the expressions of *OsACO* and *OsACO3* in germination, the inhibited germination rate and promoted coleoptile growth could not be recovered in the *OsNAC2*-overexpressing lines (Figure 4), probably due to the enhanced the biosynthesis of ABA. Thus, our finding partly evidenced that the inhibition of ABA on the germination of seeds is apparently not regulated by enhanced the biosynthesis of ethylene. In other words, the promoted effect of ethylene in the germination of seeds might be reversed by the excessive accumulation of ABA.

It has been well characterized that transcription factors modulate multiple processes in plant growth and stress response with the use of different downstream components. At present, a growing number of reports demonstrate that a single transcription factor could target two or more downstream genes. TILLER INCLINED GROWTH 1 (TIG1) directly binds to the promoters of *SAUR39*, *EXPA3*, and *EXPB5* to accelerate cell elongation and enhance the tiller angle (Zhang et al., 2019). A rice HD-Zip transcription OsTF1L targets promoters of poxN/PRX38, Nodulin protein, DHHC4, CASPL5B1, and AAA-type ATPase, which are involved in the biosynthesis of lignin and drought response (Bang et al., 2019). OsbZIP62 could bind to the promoters of several putative target genes, e.g., *ZTP2*, *ZTP3*, and *ZTP4*, to enhance drought and oxidative resistance in rice (Yang et al., 2019). However, whether a transcription factor modulates its different downstream targets depending on different plant physiological processes or in a time-dependent manner has still been rarely reported. In the earlier study, we demonstrated that OsNAC2 interacts with the promoter of *OsKO2*, key gene of GA synthesis, to restrain the height of the seedling plant (Chen et al., 2015). In addition, endogenous ABA is accumulated to accelerate leaf senescence in the OsNAC2-overexpressing lines through targeting the promoters of ABA metabolic-related genes, namely, *OsNCED3*, *OsABA8ox1*, and *OsZEP1* (Mao et al., 2017). These results confirm that OsNAC2 binds to the promoters of multiple target genes, performing different physiological functions. To our interest, during the germination period, the expressions of ABA synthesis-related genes were displayed in an OsNAC2-dependent manner, while those of *OsKO2* were displayed in an OsNAC2-independent manner, which was consistent with the contents of ABA and GA (Figure 4). Furthermore, OsNAC2 could still directly interact with the promoters of ABA synthesis-related genes in the physiological process of germination of seeds, whereas this regulatory relationship between OsNAC2 and *OsKO2* was destructed in this process by using ChIP–qPCR (Figure 5). The difference between germination and seedling periods of OsNAC2 in the transcriptional regulation of *OsKO2* suggested the spatiotemporal mechanism of the transcription factor in modulating its targets. It has been reported that phosphorylation is a key switch of transcription factor Ideal Plant Architecture 1 (IPA1) in regulating both rice yield and disease resistance (Wang et al., 2018). Further research on protein modification could help us to better understand the modulatory mechanisms of OsNAC2, targeting different downstream components.

**FIGURE 7:**
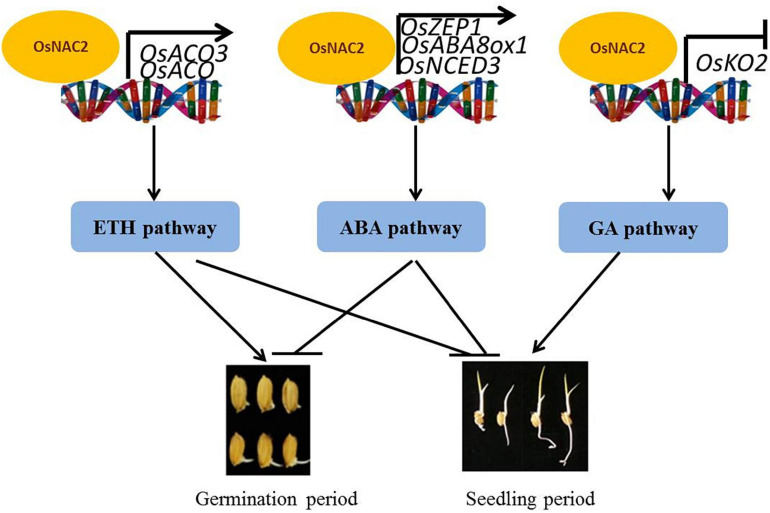
Working model of OsNAC2 in rice seed and seedling processes.

Our findings are summarized in the model shown in Figure 7. OsNAC2 is involved in multiple phytohormone pathways, including ethylene, ABA, and GA, to regulate the germination of seeds and seedling growth in rice. Specifically, OsNAC2 enhances ethylene synthesis and response by directly regulating the expressions of *OsACO* and *OsACO3*, which subsequently weakens the root growth in the establishment of seedling. In our earlier study, OsNAC2 interacts with the promoters of *OsKO2* and ABA metabolic-related genes to restrain the height of the plant and accelerate leaf senescence in rice seedling, respectively (Chen et al., 2015; Mao et al., 2017). OsNAC2 inhibits the germination of seeds probably through the ABA pathway by targeting *OsNCED3*, *OsABA8ox1*, and *OsZEP1* promoters, but not by targeting the *OsKO2* promoter, which suggested that OsNAC2 regulates its downstream targets in a time-dependent manner. Although OsNAC2 still directly increased the expressions of *OsACO* and *OsACO3*, the effect of ethylene in the germination of seeds was probably reversed by the enhanced biosynthesis of ABA. Thus, our finding enriches the regulatory network of ethylene, ABA, and GA in the germination of rice seeds and seedling growth and uncovers new insights into the difference of transcription factors in targeting their downstream components.

## Materials and Methods

### Plant Materials

Plant materials used in this study were previously generated from the *OsNAC2*-overexpressing lines (i.e., ON7 and ON11, expressing the OsNAC2–mGFP fusion protein) and the *OsNAC2*-knockdown lines (i.e., RNAi25 and RNAi31) with a *Nipponbare* genetic background (Chen et al., 2015; Mao et al., 2017). The seeds were first soaked in water at 30°C for 24 h and then transferred to water at 37°C. The experiment was conducted at least three times, with 50 seeds in each Petri dish. After germination, the rice seedlings were cultured in a phytotron with a 16/8 h light/dark cycle, 28°C, 300 W/m^2^ light intensity, and 40% humidity regime.

### Phytohormone Treatment

For the ethylene-response assay, after 48 h of normal germination, the rice seeds of five lines were transferred into the 96-well plates and were cultured in pure water containing 30 μM of ACC and pure water containing both kinds of reagents under dark conditions for 3 days. The root length and seedling length of these rice seedlings were measured, and the phenotypes were recorded by using photographing. For the treatment with ABA, GA, and their inhibitors, after soaking in water at 30°C for 24 h, the seeds were transferred to water at 37°C and treated with 10, 50, and 100 mg/L of fluridone or paclobutrazol for 48 h, and then the germination rate and the coleoptile length were observed and measured. Germination was scored as the emergence of the radicle, while seedling establishment was scored by measuring the proportion of seedlings having green cotyledons (also called sprout in this study), elongated roots, or both (Subbiah and Reddy, 2010).

### Quantitative PCR Analysis

Total RNA was extracted from the rice seedlings (ethylene-treated and untreated) and germination rice seeds (hormone-treated and untreated) with the RNAiso Plus reagent (TaKaRa, CAS No. 108-95-2). Then, total RNA was reverse-transcribed using Hifair III 1st Strand cDNA Synthesis SuperMix (Yeasen CAT: 11141ES60). The qPCR was performed using TransStart Tip Green qPCR SuperMix (TransGen Code: AQ141-04) and the MyiQ2 Real-time PCR Detection system (Bio-Rad) according to the instructions of the manufacturer. The primers used are listed in Supplementary Table 1.

### Chromatin Immunoprecipitation–Sequencing and ChIP–qPCR

ChIP was performed as described earlier (Gendrel et al., 2005; Chen et al., 2015) with seeds and seedling expressing the OsNAC2–mGFP fusion protein. For ChIP sequencing, libraries were generated by the Ovation Ultralow Library System 2 (Nugene) according to the protocol of the manufacturer. The amount of total sample should be at least 20 ng. Samples were sequenced using the paired-end 100 bp mode of the HiSeq 2000 system (Illumina). Peaks represent the areas with high sequencing read density. For ChIP–qPCR, after anti-GFP antibody immunoprecipitation, the DNA fragments were analyzed by using qPCR for three times. The primers used are listed in Supplementary Table 2.

### Yeast One-Hybrid Assay

*OsNAC2* coding sequence was constructed on the pGADT7 vector (*Eco*RI–*Bam*HI), while the promoter sequences of *OsACO* and *OsACO3* (ABA synthesis genes) were constructed on vector pHIS2.1 (*Eco*RI–*Mlu*I). The recombinant plasmids were transformed into cells of yeast strain AH109 *via* PEG/LiAc method. The empty vector was used as control. The transformed yeast cells were cultured on the SD/-Leu/-Trp/-His medium with 50 mM of 3-amino-1,2,4-triazole to screen interactions between OsNAC2 and its possible targets.

### Determination of Ethylene Content

Three-day rhubarb rice seedlings were grouped (20 in each group) into CNW with 18 mm thread mouth, 20 ml clear round-bottomed empty sample bottle; 1 ml of water was added, and the sample was covered tightly with a magnetic metal cap; after 12 h, extraction was performed with the gas chromatography for 120 s with a needle cylinder containing 0.2 ml of gas (conversion to *H* = 1/30 h), after which, numerical A (PPM), ethylene content, each sample weighing *W* (kg), air volume *V* of the sample bottle (L), were calculated. Then, the absolute content of ethylene was calculated using the formula: (*A* × *V*)/(*W* × *H*) (nl/g.h).

### Determination of ABA and GA Content

The rice seeds (RNAi25, ON11, and WT) were immersed in pure water at 30°C and 37°C, respectively, for 24 h. A total of 0.25 g seeds of different lines were taken and snap-frozen in liquid nitrogen for the measurement of ABA and GA by a third party for testing (Wuhan Greenword Creation Technology Co., Ltd.). Quantitative determination of endogenous ABA and DA was performed on a ultraperformance liquid chromatography–tandem mass spectrometry (UPLC–MS/MS) system consisting of an AB SCIEX 4500 triple quadrupole mass spectrometer and a Shimadzu LC-30AD UPLC system. Three independent biological repeats were performed.

### Tilling Mutant and Verification

The tilling mutants of *OsABA8ox1* and *OsACO* were provided by Chunming Liu Laboratory at Key Laboratory of Plant Molecular Physiology, Chinese Academy of Agricultural Sciences. The mutants were grown in a standard paddy field and then under conventional cultivation condition. For mutation verification, 1-week-old mutant leaves were used for DNA extraction by using the CTAB (hexadecyl trimethyl ammonium bromide) method. The primers for amplifying the fragments of *OsABA8ox1* and *OsACO* are listed in Supplementary Table 4. The obtained DNA fragments were sequenced by Majorbio Biotech (Shanghai, China). In addition, the seedling phenotype of *osaco* and the germination rate and coleoptile of *osaba8ox1* were also assessed.

### Accession Numbers

Sequence data from this article can be found in GenBank/EMBL databases under the following accession numbers: *OsNCED3* (Os03g0645900), *OsNAC2* (Os04g0460600), *OsABA8ox1* (Os02g0703600), *OsZEP1* (Os04g0448900), *OsActin* (Os10g0510000), *OsACO* (Os09g0570800), *OsACO3* (Os02g0771600), *OsKO2* (Os06g0570100), *OsACS1* (Os03g0727600), *OsERF1* (Os04g0546800), *OsNCED1* (Os02g0704000), *OsGA20ox1* (Os03g0856700), *OsGA20ox2* (Os01g0883800), and *OsGA20ox3* (Os07g0169700).

### Statistical Analysis

Experiments in this study, e.g., gene expressions detected by using qPCR, promoter enrichment by using ChIP–qPCR, germination rate, the length of root, coleoptile, and seedling with or without phytohormone treatment, and detection of ethylene, GA, and ABA contents were all performed at least three times. Data are presented as the mean ± standard error of at least three biological replicates, and the significant analysis was carried out with the Student’s *t*-test.

## Data Availability Statement

The datasets presented in this study can be found in online repositories. The names of the repository/repositories and accession number(s) can be found below: NCBI SRA; SRX3436029 and SRX3436028.

## Author Contributions

FM and CL conceived the project and designed the study. JY and CM conducted the main experiments and wrote the manuscript. QZ provided experimental assistance to CM and JY. FM and CM revised the manuscript. XY and CL provided the tilling mutants. All the authors commented on the results and the manuscript.

## Conflict of Interest

The authors declare that the research was conducted in the absence of any commercial or financial relationships that could be construed as a potential conflict of interest.

## Publisher’s Note

All claims expressed in this article are solely those of the authors and do not necessarily represent those of their affiliated organizations, or those of the publisher, the editors and the reviewers. Any product that may be evaluated in this article, or claim that may be made by its manufacturer, is not guaranteed or endorsed by the publisher.

## Supplementary Material

Supplementary Figure 1 The phenotype of the sprout and root growth of the OsNAC2-overexpressing plants under 1-aminocyclopropane-1-carboxylic acid (ACC) treatment.

Supplementary Figure 2 The phenotype of coleoptile growth of different OsNAC2-transgenic plants after fluridone or paclobutrazol treatment.

Supplementary Figure 3 The germination rate and coleoptile growth of various OsNAC2-overexpressing plants after gibberellic acid (GA) treatment.

Supplementary Table 1 Sequences of the primers used for quantitative polymerase chain reaction (qPCR).

Supplementary Table 2 Sequences of the primers used for chromatin immunoprecipitation –quantitative polymerase chain reaction (ChIP–qPCR).

Supplementary Table 3 Sequences of the primers used for yeast one–hybrid assay.

Supplementary Table 4 Sequences of the primers used for mutant verification.
