# Supplementary material for: OsNAC2 Is Involved in Multiple Hormonal Pathways to Mediate Germination of Rice Seeds and Establishment of Seedling
Source: Front Plant Sci. 2021 Jul 23;12:699303. doi: 10.3389/fpls.2021.699303 (PMC8343022; doi:10.3389/fpls.2021.699303)
Supplement: Supplementary Figure 1 — The phenotype of the sprout and root growth of the OsNAC2-overexpressing plants under 1-aminocyclopropane-1-carboxylic acid (ACC) treatment. [file Data_Sheet_1.docx]

**Supporting Information**

**
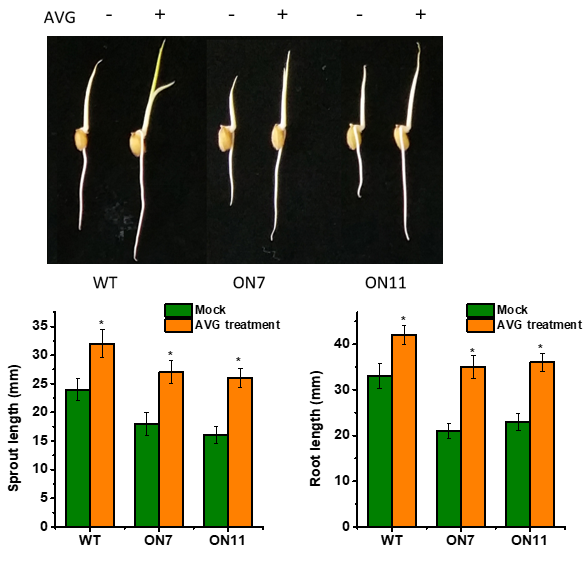
**


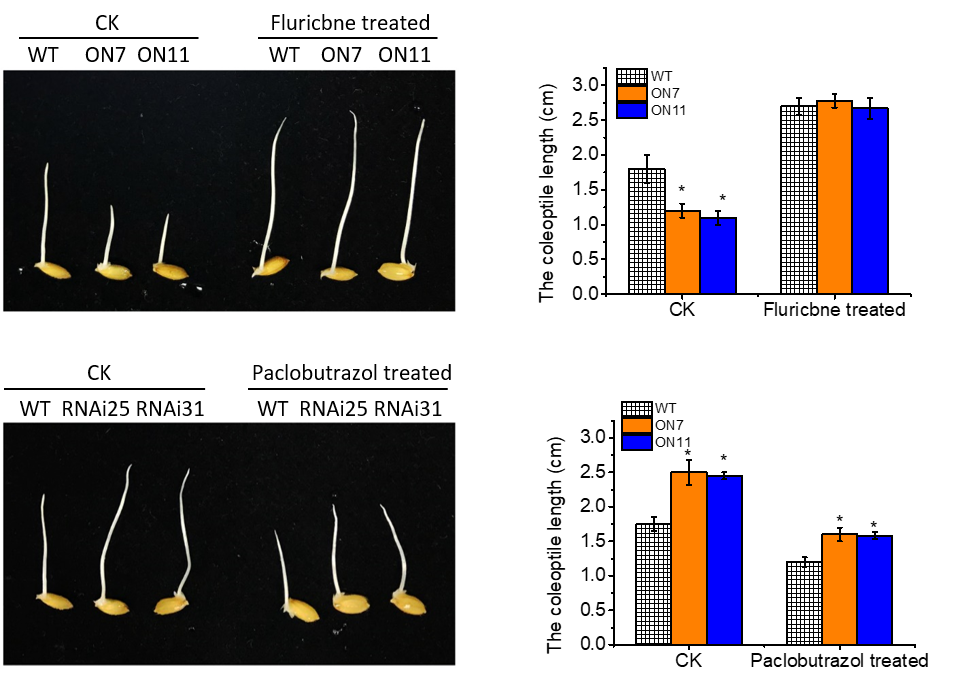
Figure S1 The phenotype of sprout and root growth of *OsNAC2*-overexpressing plants under ACG treatment.

Figure S2 The phenotype of coleoptile growth of different *OsNAC2*-transgenic plants after fluricbne or paclobutrazol treatment.


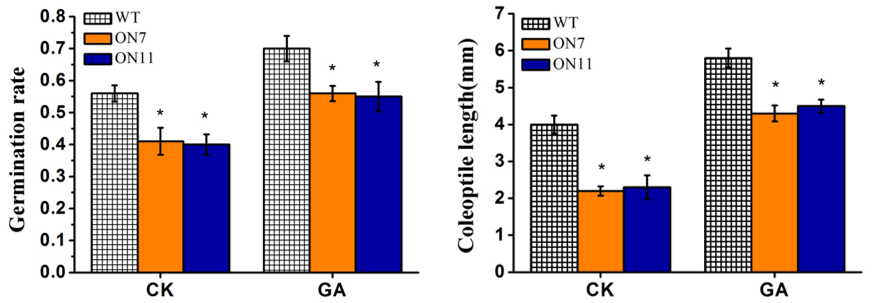


Figure S3 The germination rate and coleoptile growth of different *OsNAC2*-overexpressing plants after GA treatment.

Table S1 Sequences of the primers used for quantitative real-time PCR

| Primers | Sequences |
| --- | --- |
| OsActin-F | CTGCGATAATGGAACTGGT |
| OsActin-R | ACAATGCTGGGGAAGACA |
| OsNAC2-F | GAGAAGTCTGGCTGGGTCAT |
| OsNAC2-R | AACACCCACTCGTTGTTGGA |
| OsACO-F | TCCGACTACGGCCTGCTA |
| OsACO-R | GCTCACCTCCAGCCCTCT |
| OsACO3-F | GAGGTTCGTGTTCGAGGACT |
| OsACO3-R | CGCAGCCGTAGCTAGTGAAG |
| OsACS1-F | ACAAAACCACACCATGTCCA |
| OsACS1-R | CGAAAGGAATCTGCTACTGCTGC |
| OsERF1-F | ACCTCGGAGTCGTCCTTCTC |
| OsERF1-R  OsEIL1-F  OsEIL1-R  OsNCED1-F  OsNCED1-R  OsNCED2-F  OsNCED2-R  OsNCED3-F  OsNCED3-R  OsZEP1-F  OsZEP1-R  OsKO2-F  OsKO2-R  OsABA8ox1-F  OsABA8ox1-R  OsGA20ox1-F  OsGA20ox1-R  OsGA20ox2-F  OsGA20ox2-R  OsGA20ox3-F  OsGA20ox3-R | CGCCTCTCTTTCTCCGTTT  AGATCGGCAGGGAGAGGT  CAACGCACAACCTCGAAA  TCCATGAGGCTTTCCTACATC  GCGTTCTTCTTCCTGCCATA  CTTCCCCAGCGAACACAC  AATGAGAAGGGAGTGGAGCAT  CAAAACCCACGCGATTGTA  AGCATATCCTGGCGTCGT  CGCTGCAGCAGATTCTTG  CAACAACATGGCTATCGTTCA  CAAGAACCCTGACAAACAGGA  GAGGTGCTCCTCGGTGAC  AACACCTTCCAAGAGATGAAGACT  CTCCAGCGTCAGGTAGCACT  AGACCGGCGCCACTACTT  AGTTGAGGCGCATGATGG  CACGGGTTCTTCCAGGTTAC  GGAGTTCCATGATCGTCAGC  CGAGGAATACCGCCACAT  GAGCACCTCCGTCACGTC |

Table S2 Sequences of the primers used for ChIP-qPCR

| Primers | Sequences |
| --- | --- |
| OsACO-F1 | AAATGCTTTGTAGAATGTGAAT |
| OsACO-R1 | ATACTTAAAAAGGTTGGGTCAA |
| OsACO-F2 | ATGTTTGGTTTGAGGGGCTAGA |
| OsACO-R2 | TCAATATTTGTTAGGTGTTTTA |
| OsACO-F3 | TAATTATGGACCGTGCCTTTAG |
| OsACO-R3 | TACATGTGTCATTGACCCCTTC |
| OsACO-F4 | ACCCCCTCCCTATACTCTTCCC |
| OsACO-R4 | GGAAGTGGATATAAGCCTGCGT |
| OsACO-F5 | CATCGTCGGGCCACCGCCTCCC |
| OsACO-R5 | TATACAATTCAATTTGTGCTTC |
| OsACO-F6 | ATGTTAGGAATTTACAAGATTT |
| OsACO-R6 | CGAAGCCTCACCCATTCTAATT |
| OsACO-F7 | GTTGTGAAAAATTCTAAAAAAA |
| OsACO-R7 | ATTCAGCCTATGAATAGTAGGG |
| OsACO3-F1 | ATATGAAATTAAAACAGGTCCA |
| OsACO3-R1 | CAATGACGACTAACGTTGATAT |
| OsACO3-F2 | AAATCGAAGTGACGAAGCTCGG |
| OsACO3-R2 | CTAGCGTATGTTCTTTTTCTTT |
| OsACO3-F3 | CAGTTGGTCCGGAGATCGTCAG |
| OsACO3-R3 | TGATGAACCCTAATTAGTTATT |
| OsACO3-F4 | GAACCGGTTTCTATATTTCTAG |
| OsACO3-R4 | ACAAACGTGCCCTGGCCTATAC |
| OsACO3-F5 | TCGGGCGATCCCCAAAACCGGC |
| OsACO3-R5 | TGTACATGCATCAATGCATGCA |
| OsACO3-F6 | CAACCAATCGTTGGGACTGGGG |
| OsACO3-R6 | CAGTTGAAAACTCTTGAGGCTT |
| OsABA8ox1pro-1F | ACCGATCAGTTGATTAATCCGGCT |
| OsABA8ox1pro-1R | CTGAGTTTCCCGGTTTCTCCTCCG |
| OsNCED3pro-6F | AATCCAGTTGGCACCACAGGACTC |
| OsNCED3pro-6R | TACTACACTTAAAAACTTTGTCTC |
| KO2pro-F2-F | GTGTAGTGCTCATTAATCAGGA |
| KO2pro-F2-R | GTGTAGTGCTCATTAATCAGGA |

Table S3 Sequences of the primers used for yeast one hybrid

| Primers | Sequences |
| --- | --- |
| OsACO-*Eco* R I-F | CGgaattcATCAACGTTAGTCGTCATTGAAATC |
| OsACO-*Mul* I-R | CGacgcgtCGGAGGAGAGAAGAGGAAGAG |
| OsACO3- *Eco* R I-F | CGgaattcCTTAGATTGTGTGGATATATAGG |
| OsACO3- *Mul* I-R | CGacgcgtTGGGAGTGCGTCTGGCTTGCTTAAC |

Table S4 Sequences of the primers used for mutant verification

| Primers | Sequences |
| --- | --- |
| aba8ox1-F | CTAACTCCTCTTCCCACTCTGCTT |
| aba8ox1-R | ATTTCAGTTAAGGAACCAGTCTGC |
| aco-F | AAGCCAACATCGGCGAAGGCTCAG |
| aco-R | ATGAGCTCCCGGAACAGCCTCTGG |
